# Harnessing Evolutionary Toxins for Signaling: Reactive Oxygen Species, Nitric Oxide and Hydrogen Sulfide in Plant Cell Regulation

**DOI:** 10.3389/fpls.2017.00189

**Published:** 2017-02-10

**Authors:** John T. Hancock

**Affiliations:** Department of Applied Sciences, Faculty of Health and Applied Sciences, University of the West of EnglandBristol, UK

**Keywords:** ammonia, evolution, hydrogen sulfide, nitric oxide, reactive oxygen species, redox signaling, signal transduction

## Abstract

During the early periods of evolution, as well as in niche environments today, organisms have had to learn to tolerate the presence of many reactive compounds, such as reactive oxygen species, nitric oxide, and hydrogen sulfide. It is now known that such compounds are instrumental in the signaling processes in plant cells. There are enzymes which can make them, while downstream of their signaling pathways are coming to light. These include the production of cGMP, the activation of MAP kinases and transcription factors, and the modification of thiol groups on many proteins. However, organisms have also had to tolerate other reactive compounds such as ammonia, methane, and hydrogen gas, and these too are being found to have profound effects on signaling in cells. Before a holistic view of how such signaling works, the full effects and interactions of all such reactive compounds needs to be embraced. A full understanding will be beneficial to both agriculture and future therapeutic strategies.

## Introduction

Reactive compounds such as those derived from oxygen, nitrogen, and sulfur are instrumental in cell signaling pathways (Mittler et al., 2011; Mur et al., 2013; García-Mata and Lamattina, 2013; Hancock and Whiteman, 2014). It appears that they have effects in a wide range of organisms from simple prokaryotes to humans and higher plants. However, despite the fact that organisms are using such compounds in a positive way, this use belies their inherent toxic nature. It appears therefore that during evolution cells have had to tolerate the presence of such compounds and have over time adopted them for their own gains.

The atmosphere during the history of the Earth has not been unchanging. Four billion years ago the atmosphere would have been approximately one part per million oxygen (Lane, 2002) and yet today many organisms easily survive in 21% oxygen (over 200,000 parts per million). Approximately two and half billion years ago oxygen would have started to increase due to biological activity (Lyons et al., 2014). It would not have been a sudden rise but as organisms evolved they had a new toxin to contend with. Oxygen is a di-radical (Cheeseman and Slater, 1993) and undergoes redox reactions to yield a family of reactive compounds [the so called reactive oxygen species (ROS)], including the superoxide anion, hydrogen peroxide (H_2_O_2_) and the hydroxyl radical. The issue for newly evolving organisms as oxygen levels rose was that many of the ROS are toxic (Wallace and Melov, 1998; Halliwell and Gutteridge, 2015). Therefore to counter this organisms have evolved a wide range of antioxidant defenses, which prevents the build-up of ROS and limits the damage that may be done (Blokhina et al., 2003). These include enzymes such as superoxide dismutase (SOD) and catalase, as well as small compounds such as ascorbate and glutathione (GSH). Manipulation of these, such as levels of SOD, has been shown to increase life span in some species (Parkes et al., 1998) showing that the control of ROS levels is crucially important. Furthermore, in a treatise on glutathione levels Schafer and Buettner (2001) discussed the importance of the maintenance of intracellular redox status – it must be kept much reduced – and how oxidation can lead to either apoptosis or necrosis. However, despite all this, cells still use ROS as signaling molecules (Mittler et al., 2011). It appears that the presence of oxygen in the atmosphere has had a profound influence in the evolution of aerobic organisms, as has been discussed by others (Lane, 2002; Dowling and Simmons, 2009; Metcalfe and Alonso-Alvarez, 2010). During such evolution cells have not just learnt to tolerate the presence of oxygen and its downstream products, but have harnessed such products for a positive action; both in and between cells.

Similar tolerance of toxic compounds can be seen with nitrogen- and sulfur-based compounds. The most commonly studied compound here is nitric oxide (NO). This was found to be instrumental in the control of vascular tone in mammals, where it was originally known as endothelial-derived relaxing factor (EDRF: Palmer et al., 1987) but has since been found to be a key part of cell signaling in a range of organisms including plants. Exposure of plants to NO can be from natural sources such as the soil (Davidson, 1991; Skiba et al., 1993; Ludwig et al., 2001). Plants also have the capacity to make intracellular NO (reviewed by Mur et al., 2013). However, NO is inherently toxic, and for animals diet may help here, showing that plants cells have compounds which mitigate against the harmful effects of this compound (Paquay et al., 2000). Peroxynitrite, derived from the reaction of NO with ROS is also toxic (Bartosz, 1996) but it is also known to be involved in signaling (Klotz, 2005).

Evolution has also been shaped by the presence of hydrogen sulfide (H_2_S). H_2_S is produced at thermal vents (Martin et al., 2008), where many organisms still rely on the presence of sulfur compounds as a source of reducing power. While many organisms have adapted to life in the presence of H_2_S (Tobler et al., 2016), such as fish in H_2_S-rich springs (Kelley et al., 2016), clearly life also has left such niche environments. Therefore during evolution species have developed, some remaining in the presence of, and tolerating, H_2_S while others has escaped it into an oxygen-rich environment. H_2_S is, like other reactive compounds considered here, very toxic. It is known, for example, that H_2_S is an inhibitor of mitochondrial electron transport chains (Complex IV) and so inhibits ATP production (Dorman et al., 2002). It is so toxic that it was used as a chemical weapon (Szinicz, 2005), yet organisms have harnessed it as a signaling molecule. It has shaped events in evolution and been adopted as part of metabolism (Olson and Strub, 2015). Bacteria are known to produce H_2_S (Clarke, 1953), in plants H_2_S is used in sulfur metabolism (Calderwood and Kopriva, 2014), whilst at very low concentrations in animals instead of inhibiting the electron transport chain of mitochondria it has been shown to be a source of reducing power for the production of ATP (Bouillaud et al., 2013). Here is a good example of how organisms have evolved in the presence of a toxic compound but adapted to use it for positive reasons.

The majority of the literature regarding the signaling by reactive compounds concentrates on ROS, NO, and most recently H_2_S. However, the early atmosphere of the Earth’s history was also rich in other noxious compounds, such as methane, ammonia, and hydrogen (Lane, 2002). Such compounds should also be included in the suite of potential cell signaling molecules, giving a more holistic understanding of how all these compounds may be controlling cellular functions in plants.

### Roles of Reactive Signaling Compounds

Signaling in cells involves a myriad of different components, some of which are small transient molecules. When a molecule has been proposed as a signaling component there are certain criteria that may be looked for. It should be made where and when needed, be recognized as being present (so it may transmit a specific message), be able to move the message to a new position in the cell (or to another cell), and be removed when no longer needed (Hancock, 2016). Looking at ROS, NO, and H_2_S it can be argued that such criteria are met.

Enzymes are involved in the generation of reactive signals. As such proteins are often only active when required and usually have defined subcellular locations, the reactive molecules produced are only present where and when needed. ROS are generated from the NADPH oxidase family of enzymes, but enzymes such as peroxidases may also contribute to ROS production. There is some controversy about the production of NO in plants. There is almost certainly no nitric oxide synthase (NOS) in higher plants (Jeandroz et al., 2016) but plants can generate NO from other enzymes such as nitrate reductase (Rockel et al., 2002). H_2_S can be generated by desulfhydrases in plants (Alvarez et al., 2010). Removal of ROS will be through antioxidants whilst NO will react with thiols, metals or be oxidized. H_2_S can be removed through the action of *O*-acetylserine (thiol) lyase (Youssefian et al., 1993).

ROS, NO, and H_2_S are all diffusible so they are all able to move their message through, or between, the cells. However, some care is needed when discussing if membranes can be traversed. For example, NO can be a radical and uncharged but the loss or gain of an electron will yield NO^+^ and NO^-^; both are hydrophilic. In a similar manner, the ROS H_2_O_2_ is neutral and can move across the lipid bilayer but $O_{2}^{•–}$ would not, unless protonated. Furthermore, it must be considered that such compounds can react with the membranes themselves, leading to lipid peroxidation or the formation of nitro-lipids. The formation of nitro-fatty acids has been suggested to be important for further signaling (Mata-Pérez et al., 2016).

It can be seen, therefore, that ROS, NO, and H_2_S can partake in signaling, that is, so long as their concentrations do not rise to toxic levels. One of the common themes of their use in plants is in response to stress (Misra et al., 2011; Petrov and Van Breusegem, 2012; Hancock and Whiteman, 2014). The list of stresses investigated in plants in which such signaling is implicated is wide ranging and includes: water stress; salt stress; pathogen challenge; heat/cold stress; metal ion (for example cadmium, copper, aluminum) stress. Under stress conditions the production of ROS etc is increased and this often impacts on the expression of antioxidant systems. However, ROS, NO, and H_2_S are also involved in normal plant development and function, such as: germination (Dooley et al., 2013); root development (Osuna et al., 2015); stomatal closure (Lisjak et al., 2010; Murata et al., 2015); flower senescence (Zhang et al., 2011).

In order for ROS, NO, and H_2_S to be involved in signaling, once they are produced their presence has to be perceived for the message transduction to continue. With NO, the classical pathway determined in animals is the activation of the enzyme guanylyl cyclase and the resultant increase in cytosolic cGMP concentrations. Similar pathways have been studied in plants (Gross and Durner, 2016). However, one of the main mechanisms by which these reactive compounds participate in signaling is through the modification of the thiol groups of proteins. Thiol groups can be oxidized, as was seen with glyceraldehyde 3-phosphate dehydrogenase (GAPDH: Hancock et al., 2005), nitrosated (Lindermayr et al., 2005) or *S*-sulfhydrated (Sen et al., 2012; Romero et al., 2013). In each case the thiol group will be covalently modified in a reversible manner (although some modifications such as the formation of the sulphonic acid group seems to be irreversible), in such a way that the protein may have an altered function, as would be needed for signaling. This is akin to phosphorylation/dephosphorylation. Therefore, through such actions the signal can be transduced to the next component of the pathway leading to the appropriate cellular response.

### Interactions of Reactive Signaling Compounds

It is wrong to think about ROS, NO, and H_2_S working in isolation from each other. As mentioned above, reactions can take place between them. Superoxide anions and NO can react to form peroxynitrite, a possible signaling molecule (Klotz, 2005). NO and H_2_S can react to create nitrothiols, again with signaling potential (Whiteman et al., 2006), whilst ROS and H_2_S can also create downstream products (Li and Lancaster, 2013). It is known that NO and H_2_S can affect antioxidant levels in cells, and so influence ROS signaling. For example H_2_S will increase glutathione generation (De Kok et al., 1985), while others report alterations in ascorbate and antioxidant-related enzymes following H_2_S treatment (Shan et al., 2011). On the other hand, the activity of glucose-6-phosphate dehydrogenase (G6PDH) was increased following H_2_S treatment, which may increase ROS accumulation (Li et al., 2013). Therefore there will be interplay between such signaling molecules (Hancock and Whiteman, 2014, 2015). Either they can influence each other’s generation, or they can scavenge each other, lowering the intracellular concentrations to reduce, or nullify, their effects.

As discussed above, thiols can be modified by this suite of reactive signaling molecules but of course they may be in direct competition with each other. Some proteins, such as GAPDH are known to be modified by both ROS and NO (Hancock et al., 2005), and this will not be the only competitive target. Furthermore, other convergence points may exist. It is known, for example, that the activity of MAP kinases are influenced by both ROS and NO (Kovtun et al., 2000; Wang et al., 2010) and it would be no surprise to find H_2_S having a similar effect.

## Conclusion and Future Directions

It is clear therefore that during evolution certain molecules to which organisms have been exposed have not simply been tolerated but that they have been adopted as part of the suite of chemicals used for signaling. The most studied of these are ROS such as hydrogen peroxide (Mittler et al., 2011), NO (Mur et al., 2013), and H_2_S (Hancock and Whiteman, 2014). It may be that as such molecules had to be removed low levels always remained, while removal processes automatically gave cells a way to reverse cell signaling processes involving these compounds. What is clear is that carefully controlling the intracellular, and in some cases extracellular, concentrations of these reactive molecules are crucial for cell survival. Too much and crucial enzymes are inhibited, such as cytochrome oxidase (Dorman et al., 2002), or cellular damage ensues such as lipid peroxidation and DNA damage (Jena, 2012). Fluctuate the concentrations within defined limits and signaling can safely take place. Compartmentalisation is important here and may be part of the key to understanding how these signaling systems work without causing intolerable damage.

Besides ROS, NO, and H_2_S the early atmosphere of the Earth contained other small relatively reactive compounds. Amongst these are ammonia, methane and hydrogen (Lane, 2002). Therefore it is possible that as cells had to tolerate these too, that they also have been harnessed as signaling molecules.

It is known that nitrogen reduction, for example to ammonia, was involved in the development of the atmosphere (Brandes et al., 1998). Ammonia has been shown to have effects in biological systems, amongst which is its toxicity (Britto and Kronzucker, 2002). Plants are exposed, generate and translocate ammonium (Schjoerring et al., 2002). Therefore it could be ideal as a signaling molecule. In human cells ammonium has been shown to trigger autophagy (Eng et al., 2010), where the ammonium was derived from the deamination of glutamine by glutaminolysis. Astrocyte dysfunction mediated by ammonium involved interactions with antioxidants, oxidative stress and MAP kinases (Jayakumar et al., 2006). The same group reported that ammonium induced Ca^2+^ increases in cells and suggested that this could lead to the synthesis of NO and ROS, and would involve proteins such as NAPDH oxidase, NOS, phospholipase A_2_ and NF-κB (Norenberg et al., 2009). Therefore ammonium was acting on pathways in a similar way to other reactive compounds.

Methane has been shown to alter bowel contractile movement (Pimentel et al., 2006). The methane in this case was produced by bacteria in the gut flora. Another compound which may need to be considered is sulfur dioxide, which has been shown to reduce the proliferation of smooth muscle cells through a mechanism which involves MAP kinases and cAMP signaling (including activation of cAMP-dependent protein kinase: Liu et al., 2014). Both these compounds therefore impinge on signaling in animals.

A molecule that has had a lot of recent interest in signaling is hydrogen gas. In animals for example, in a study on ischemia/reperfusion injury of liver, hydrogen gas was found to activate the NF-κB pathway (Zhang et al., 2015). This seems to be a convergence point of several of these signal transduction pathways, being implicated in ROS signaling (Morgan and Liu, 2011), NO signaling (Arias-Salvatierra et al., 2011) and H_2_S effects (Sen et al., 2012). In plants hydrogen gas has been found to be involved in a range of stress responses, just as seen with ROS, NO, and H_2_S. Zhu et al. (2016) in the introduction of their paper lists salt stress, toxicity of metals such as cadmium, aluminum and mercury, and oxidative stress. They go on to say that hydrogen gas inhibited NO production in animals (Itoh et al., 2011), and then showed that in plants hydrogen gas-induced generation of adventitious roots required NO in the downstream signaling cascades (Zhu et al., 2016). Therefore, as with the other reactive compounds discussed above, hydrogen gas impinges on these signaling systems and should be considered along with the other reactive molecules for a full understanding of signaling in plants.

Lastly, it is noteworthy that the understanding of how some of these reactive signals are working may have practical implications. It has been suggested that H_2_S and hydrogen gas may slow fruit ripening and senescence (Hu et al., 2012, 2014), while in animal research H_2_S has been mooted as an important future therapeutic agent (Zhang et al., 2013). Low levels of such compounds have even been shown to increase life-span in some organisms (Miller and Roth, 2007), despite their inherent toxicity.

In conclusion, there has been much interest in how ROS, NO, and H_2_S are used as signals in cells, including in plants. They have been tolerated and harnessed during evolution but there are other reactive compounds which need to be embraced into this suite of signaling compounds, along with the interactions which take place between them, before it can be fully understood how this signaling works. Dysfunction of such signaling can have catastrophic results, while prudent use of some of these compounds may be of an advantage to future agriculture and therapeutics.

## Author Contributions

This is an invited inaugural paper as I was invited to be Associate Editor of Plant Physiology (specialty section of Frontiers in Physiology and Frontiers in Plant Science). I have written a mini-review with an opinion build in so have suggested that it should be a Perspective – hope this is correct. I am the sole author.

## Conflict of Interest Statement

The author declares that the research was conducted in the absence of any commercial or financial relationships that could be construed as a potential conflict of interest.
